# Three-Dimensional Reproductions for Surgical Decision-Making in the Treatment of Recurrent Patella Dislocation

**DOI:** 10.1016/j.eats.2023.02.010

**Published:** 2023-05-01

**Authors:** Kristin E. Yu, Brian Beitler, Daniel R. Cooperman, David Frumberg, Christopher Schneble, William McLaughlin, John P. Fulkerson

**Affiliations:** Yale School of Medicine, Department of Orthopaedics & Rehabilitation, New Haven, Connecticut, U.S.A.

## Abstract

Patellofemoral instability may be attributed to a variety of soft tissue and osseous factors, of which dysplasia of the femoral trochlea significantly predisposes patients to recurrent instability events. Surgical planning and decision-making remain wholly predicated upon two-dimensional imaging-derived measurements and classification systems, although aberrant patellar tracking in the setting of trochlea dysplasia is a three-dimensional (3-D) complexity. 3-D reconstructions of the patellofemoral joint (PFJ) may be considered to better comprehend the complex anatomy of patients with recurrent patella dislocation and/or trochlea dysplasia. We describe a classification and integrated interpretation system by which these 3-D reproductions of the PFJ may be analyzed to enhance surgical decision making in the treatment of this condition to achieve optimal joint stability and long-term preservation.

## Introduction

Two-dimensional (2-D) imaging is limited in its ability to adequately capture the critically important initial patellofemoral joint (PFJ) articulation.^1^, ^2^, ^3^, ^4^, ^5^, ^6^ 3-D reconstructions—whether printed or virtual—enhance conceptualization of aberrant PFJ anatomy and articulation in the setting of recurrent instability, particularly when complicated by trochlea dysplasia.^2^^,^^7^ Guidelines for model analysis, interpretation, and application to preoperative planning in the context of recurrent patella dislocation have yet to be devised, and the reliability of commonly used radiographic measurements and classification schemes remains a topic of debate.^8^^,^^9^ Herein, we describe our experience interpreting 3-D PFJ models from patients with recurrent patella dislocation compared to a cohort of subjects with no history of patella instability and potential implications for surgical planning.

### Technique

Three-dimensional prints were generated from patients with recurrent patella dislocation seen and treated by the senior author between January 2020 and November 2021. All patients with a history of recurrent, atraumatic patella dislocation for whom a CT scan of the knee was obtained were included. Exclusion criteria included congenital deformity and a history of trauma to the knee or arthritic disease, leaving 24 patients (18 female, 6 male) for inclusion. All patients consented to 3-D printing of his or her knee.

Three-dimensional (3D) prints were generated from 10 control subjects, all female, with no history of patella dislocation whose whole-body CT scans were included in the New Mexico Decedent Image Database. This data set comprises whole-body CT scans from more than 15,000 New Mexicans who died between 2010 and 2017, with associated metadata on 60 variables, including demography and cause of death. These control subjects were selected with respect to age-matching and atraumatic causes of death not involving damage to the knee joint. Bilateral knees were segmented and printed for each control subject, yielding a total of 20 knees.

Weight-bearing CTs were obtained using the Carestream Onsight 3D Extremity System (Carestream Health, Rochester, NY). Imaging acquisitions are obtained with the patient in full extension and 20° of knee flexion. Contrast material was not administered. CT settings included 0.625-mm slice thickness, 120 kVp tube voltage, spiral pitch ratios of 0.5, and tube current value of 150 mA.

CT DICOM files were loaded onto a medical visualization application (Simpleware ScanIP, Synopsys, Mountain View, CA) to render a 3-D volumetric data set. Segmentation of the distal femurs was performed via thresholding, edge detection, and manual segmentation. The entirety of the distal femoral metaphysis and femoral condyles were included. Stereolithography files were generated from each segmented data set. ScanIP-generated models were oriented for printing in PreForm (Formlabs, Somerville, MA), and then printed 100% to size on a Form 3BL printer (Formlabs) in Grey v4 resin at a resolution of 0.1 mm. Following the completion of printing, the model is removed from the printer and washed in isopropyl alcohol for 20 minutes in a Form Wash (Formlabs), allowed to dry, and cured via exposure to ultraviolet light. Models were analyzed as they rested on their posterior femoral condyles.

### Interpretation

**Fig 1:**
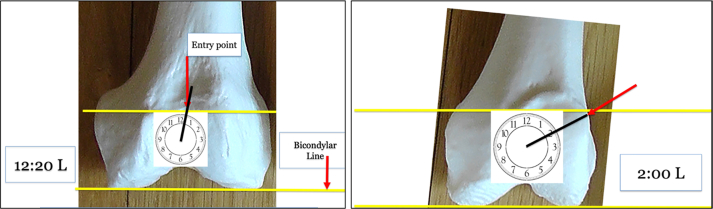
Clockface description scheme of trochlear entry points in 3D-printed knee models from a control subject with no history of patella dislocation (left) and in a patient with recurrent patella dislocation (right), both left knees. Subjects with no history of patella dislocation had entry points closer to the midline, 12 o’clock position, and smaller, more acute angles between the midline and entry point, than those with recurrent patella dislocation (i.e., negative controls). More lateralized trochlear entry points were observed with increased severity of dysplasia.

3-D reproductions enable visualization of the osseous borders to patella engagement, with the femoral trochlea with progressive knee flexion in both normal and dysplastic knees. In 3D prints, the most proximal point of the trochlear groove at which the medial and lateral trochlear facets converge likely represents the initial point of patella engagement with the femoral trochlea in early knee flexion (Video). This point lies relatively midline with respect to the axis of the distal femoral shaft in control subjects; were the femoral trochlea oriented and described relative to the face of an analog clock and centered with respect to the long axis of the femoral shaft and aligned with respect to the bicondylar axis, this point is located at ∼12 o’clock (Fig 1). This clock face analogy is rooted in the clinical manifestation of an increasingly prominent J sign on exam with knee extension. By contrast, in 3-D models from patients with recurrent patella dislocation, this point along the proximal trochlea was observed around 2-3 o’clock in left knees and 9-10 o’clock in the right knees.

**Fig 2:**
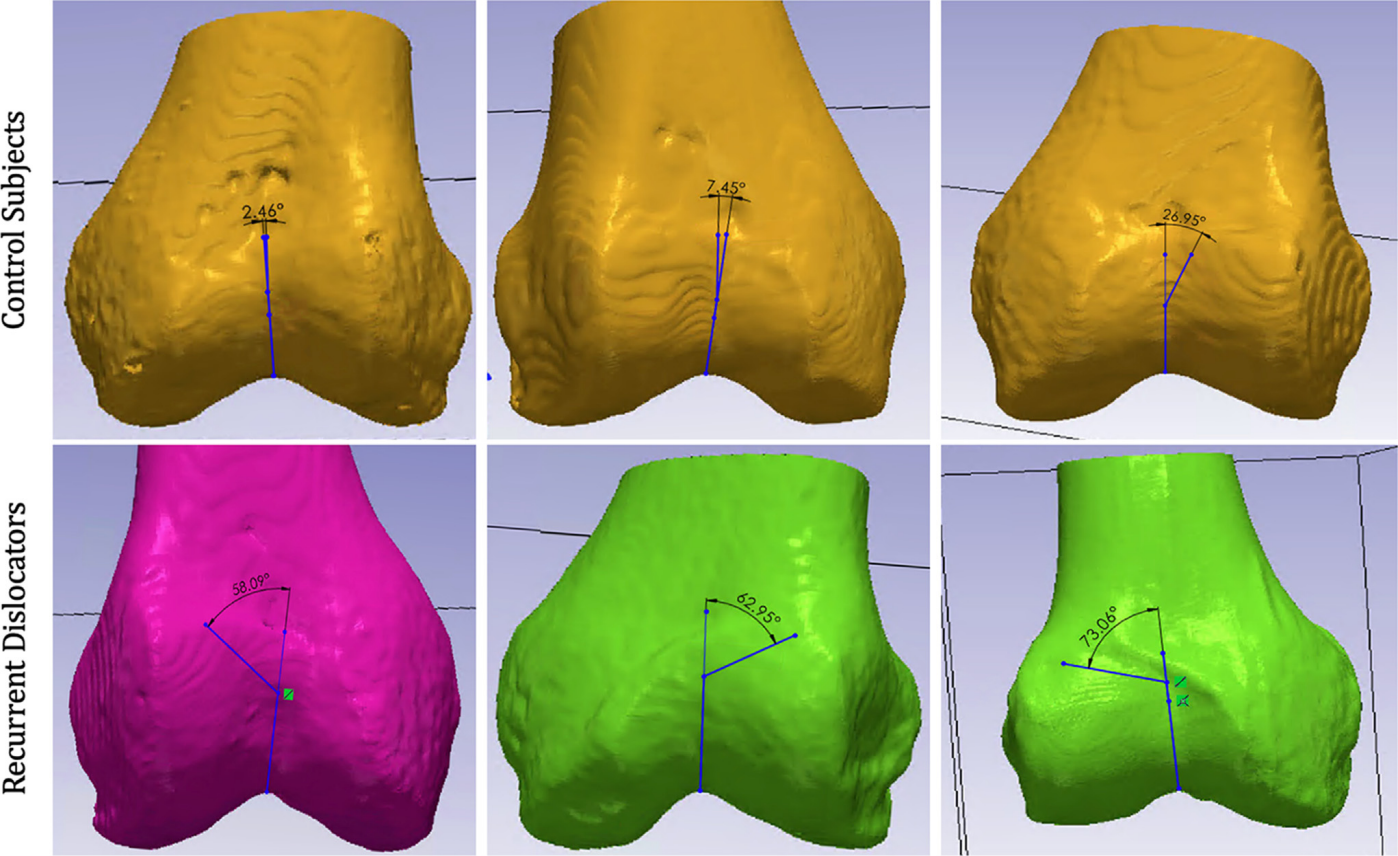
Digital 3D reconstructions of the distal femurs of subjects with no history of patella dislocation and instability (i.e., controls, top row) and those with recurrent patella dislocation (bottom row). With the reproductions “lying” flat on their posterior condyles and using the midshaft of the femur as a reference point, larger, more obtuse angles associated with more lateralized trochlear entry points were observed in the recurrent patella dislocation patients compared to the controls. According to the “clockface” classification scheme of trochlear entry point lateralization, these models are shown in order of increasing dysplasia severity from left to right for both sample rows in the figure. Larger angles between the midline of the femoral shaft and entry point in subjects with increasing severity of dysplasia.

None of the control subjects had a proximal trochlear engagement point >30° from the 12 o’clock position (mean = 23.0° from midline, SD = 5.5°; Fig 2). By contrast, lateralized patella entry points into the proximal trochlea were observed in the 3-D models of recurrent patella dislocation patents that approached the 3 and 9 o’clock positions (mean = 56.6°, SD = 18.3°; Fig 2). The position of this proximal trochlea engagement point along the “clockface” of the trochlea was used to characterize proximal trochlea dysplasia. Knees with entry points with <30° deviation from the midline, 12 o’clock position were considered relatively normal in proximal trochlea morphology; those with lateralization of the entry point by 30-45° or ∼1 hour on the clock face (i.e., 1 or 11 o’clock, depending on laterality), mildly dysplastic; those with lateralization of the entry point by 46-59° or ∼2 hours (i.e., 2 or 10 o’clock), moderately dysplastic; and those with lateralization of >60° or nearly 3 hours (i.e., 3 or 9 o’clock), severely dysplastic. No patients with recurrent patella dislocation were classified as normal to mildly dysplastic using this scheme.

The clockface scheme may be integrated into larger descriptions of trochlear groove curvature, which have implications for clinical practice in the setting of trochlea dysplasia. We draw attention to the significance of the proximal medial trochlear ridge—commonly referred to as a supratrochlear spur or bump in two-dimensional (2D) imaging-based classification systems—in defining the osseous contours of the trochlear groove within which patella engagement is constrained. The morphology of this proximal medial trochlear groove becomes increasingly oblique with progressive 3D trochlear “flatness,” yielding a sharp lateral to medial trochlear groove course for patella tracking in early knee flexion (Video 1). The appearance of the proximal medial trochlear ridge can be considered alongside the extent to which the proximal trochlear entry point is lateralized (Table 1) to determine which form of operative management is most suitable, as it is this proximal trochlea articulation during early knee flexion that is critical to overall patellofemoral joint stability. This is particularly important in patients with complex, multifactorial presentations of patella instability, such as those with neuromuscular disease (Video 1).

**Table 1:** Pearls and Pitfalls

Pearls	Pitfalls
Pay attention to the variable morphology of the proximal trochlea using 3D reproductions, as this cannot be seen on normal CT scans.	High-quality segmentation and image quality are needed to ensure accuracy of 3D models.
Orient 3D-printed models on their respective posterior femoral condyles while viewing to standardize measurements and interpretation.	In the case of severe dysplasia, the posterior femoral condyles may also be dysplastic, which may skew model interpretation when viewed from above.
When using the clockface scheme, the analog clock position should be oriented to the long axis of the femoral shaft and bicondylar axis, with the 12 o’clock position along the midline of the femoral shaft.	3D interpretation scheme should be integrated with rather than replace existing classification schemes of trochlear dysplasia for informed surgical decision making
3D interpretation of proximal trochlear dysplasia should include characterization of trochlear entry point lateralization, medial ridge obliquity, and proximal groove shape	

CT, computed tomography; 3D, three-dimensional.

## Discussion

Three-dimensional evaluation of the femoral trochlea provides an unsurpassed overview of groove curvature and laterality of patella entrance to the trochlea that cannot be well understood in 2D. In our patella dislocation patients, the entry points into the trochlea are more lateral than in control knees, resulting in sharply curvilinear courses of patellar tracking from lateral to medial with progressive knee flexion. We define the entry point for the patella as a midpoint between the proximal end points of the medial and lateral trochlear ridges. This increase in trochlear groove obliquity with greater severity of dysplasia has important potential implications in patellofemoral instability, pain, and premature arthritis in patients with trochlea dysplasia.

The most critical window of patella engagement with the trochlea in patellofemoral joint stability is within the first 30° of knee flexion.^10^ Yet this initial area of patella engagement with the proximal trochlea and how it varies between normal, asymptomatic patients and those with recurrent patella instability is impossible to understand adequately with 2D images, making clinical decision making difficult and potentially inaccurate. Three-dimensional imaging, however, provides understanding of trochlea curvature and the medial and lateral ridges^11^^,^^12^ to visualize how a patella enters and tracks in the proximal trochlea. Once one becomes comfortable identifying the patella entry point and recognizes the pattern and extent of trochlear groove curvature, one is better able to decide whether moving a patella medially, by tibial tubercle transfer osteotomy (TTO), to engage the trochlea more fully early in knee flexion will be desirable to optimize patella stability and long-term joint preservation.

Interpreting a 3D reproduction of a dysplastic knee should include careful consideration of the trochlear entry zone in surgical decision making and preoperative planning of complex patella instability patients, such as those with trochlea dysplasia and neuromuscular trochlea malformation, to optimize stability and load distributions, thereby, also reducing the risk of progression to patellofemoral arthritis. Some patients may require surgical correction involving both soft tissue reconstruction and tibial tubercle transfer osteotomy. 3D model evaluation also gives the best possible appreciation of proximal spur prominence and enables definition of the extent to which proximal medial ridge spur resection may be helpful to facilitate patella entrance to the trochlea in conjunction with tibial tubercle transfer anteromedially, medially, or distally. Three-dimensional reproductions provide a powerful guide to optimal operative management of recurrent patella dislocation and trochlea dysplasia for maximum patient benefit and joint preservation (Table 2).

**Table 2:** Advantages and Disadvantages of Three-Dimensional Modeling of Osseous Trochlear Morphology

Advantages	Disadvantages
Enables improved conceptualization of trochlear dysplasia rather than requiring extrapolation from two-dimensional images	High initial start-up cost for 3D printer, materials, and post-processing equipment, and segmentation software
Ease of interpretation with minimal learning curve for both computer-based and printed 3D models	3D representation quality, whether virtual (e.g., on computer) or physically printed, is dependent upon image and segmentation quality
Can appreciate differences in the orientation, lateralization, and shape of the trochlear groove, entry point, and medial trochlear ridge, which cannot be seen on standard CT scans	Only captures bony morphology and relationships as seen on CT scans and does not include cartilage
Unique tactile element as educational tool	

CT, computed tomography; 3D, three-dimensional.

### Limitations

The interpretation scheme described herein is by no means complete nor meant to be considered as a singular decision-making tool. This methodology should be integrated with existing classification schemes and measurement standards currently used to guide surgical decision-making for patients with recurrent patella instability and/or trochlea dysplasia (Table 2). Three-dimensional reproductions and printing involve significant start-up costs related to software, equipment, and print material acquisition, and the quality of CT scans obtained affects 3D reproduction accuracy. Moreover, this scheme was devised using a relatively small sample size of symptomatic and control subjects.

### Conclusions

Interpretation of 3D PFJ reproductions may enhance the ways by which we understand, diagnose, and approach PFJ pathology beyond what is currently possible with 2D imaging.

## Supplementary Data

ICMJE author disclosure forms

Video 1 This video uses the medial angle and a clock face to describe trochlear dysplasia. With 3-D printed models of the femoral trochlea resting on their respective posterior femoral condyles and viewed from above, the orientation of the medial trochlear ridge is seen to vary with increasing severity of trochlea dysplasia, as demonstrated by increased trochlear groove “flatness.” As such, control knees—from subjects with no history of patella instability—had sharp medial ridge angles around 90°, while increasingly dysplastic models from patients with recurrent patella dislocation had “flatter” medial ridge angles that approached 180° and were more oblique in orientation. The trochlear entry point also appears increasingly lateralized (i.e., deviates from the midline) with respect to the middle of the femoral shaft with increasing dysplasia severity. Were the position of this trochlear entry point to be described relative to a clock face, it would lie at approximately 12 o’clock in control knees and increasingly lateralize—toward the 3 and 9 o’clock positions—with increasing severity of dysplasia. Being able to conceptualize the tracking path and aberrant course of the trochlear groove is critical in the surgical management of complex patellofemoral instability, such as in patients with neuromuscular disease due to the complicated relationships between muscle tone, alignment, and trochlear morphology in this patient population, as form ultimately follows function. 3D reproductions of the patellofemoral joint may be analyzed and characterized with respect to trochlear groove lateralization and groove obliquity to enhance surgical decision making in the treatment of recurrent patellar instability to achieve optimal joint stability and long-term preservation.
